# Microarray Analysis of the Molecular Mechanism Involved in Parkinson's Disease

**DOI:** 10.1155/2018/1590465

**Published:** 2018-03-01

**Authors:** Cheng Tan, Xiaoyang Liu, Jiajun Chen

**Affiliations:** Department of Neurology, China-Japan Union Hospital of Jilin University, Changchun, Jilin 130033, China

## Abstract

**Purpose:**

This study aimed to investigate the underlying molecular mechanisms of Parkinson's disease (PD) by bioinformatics.

**Methods:**

Using the microarray dataset GSE72267 from the Gene Expression Omnibus database, which included 40 blood samples from PD patients and 19 matched controls, differentially expressed genes (DEGs) were identified after data preprocessing, followed by Gene Ontology (GO) and Kyoto Encyclopedia of Genes and Genomes (KEGG) pathway enrichment analyses. Protein-protein interaction (PPI) network, microRNA- (miRNA-) target regulatory network, and transcription factor- (TF-) target regulatory networks were constructed.

**Results:**

Of 819 DEGs obtained, 359 were upregulated and 460 were downregulated. Two GO terms, “rRNA processing” and “cytoplasm,” and two KEGG pathways, “metabolic pathways” and “TNF signaling pathway,” played roles in PD development. Intercellular adhesion molecule 1 (*ICAM1*) was the hub node in the PPI network; hsa-miR-7-5p, hsa-miR-433-3p, and hsa-miR-133b participated in PD pathogenesis. Six TFs, including zinc finger and BTB domain-containing 7A, ovo-like transcriptional repressor 1, GATA-binding protein 3, transcription factor dp-1, SMAD family member 1, and quiescin sulfhydryl oxidase 1, were related to PD.

**Conclusions:**

“rRNA processing,” “cytoplasm,” “metabolic pathways,” and “TNF signaling pathway” were key pathways involved in PD. *ICAM1*, hsa-miR-7-5p, hsa-miR-433-3p, hsa-miR-133b, and the abovementioned six TFs might play important roles in PD development.

## 1. Introduction

Parkinson's disease (PD) is one of the most common age-related neurodegenerative diseases [1]. The age at PD onset is approximately 55 years, and the incidence in the population aged > 65 years is approximately 1% [1–3]. PD mainly occurs because of the death of dopaminergic neurons in the substantia nigra [4]. Patients with PD present with symptoms such as bradykinesia, resting tremor, rigidity, and postural instability [5]. The current therapy for PD is targeted at its symptoms rather than at dopaminergic neuron degeneration [1]. The diagnosis of PD at the early stage is challenging, and successfully managing PD is difficult at its later stages [4]. To date, the cause of PD remains unknown; however, it appears to involve the intricate interplay of environmental and genetic factors [1, 4].

Much effort has been spent in investigating PD pathogenesis, and the misfolding, aggregation, and aberrance of proteins are considered to be some of the main causes [1, 4, 5]. Some key genes such as hydrogen sulfide, chromobox 5 (*CBX5*), and transcription factor 3 (*TCF3*) are related to PD [6, 7]. Several pathways have also been identified to be related to PD. Activation of the protein kinase B (Akt)/glycogen synthase kinase 3 beta/(GSK3*β*) pathway by urate reportedly protects dopaminergic neurons in a rat model of PD [8]. In addition, the E2-related factor 2 (Nrf2)/antioxidant response element pathway reportedly counteracts mitochondrial dysfunction, which is a prominent PD feature [9]. The ubiquitin, lipid, nigrostriatal, autophagy-lysosome, and endosomal pathways are also involved in PD [10–15]. Furthermore, a recent study revealed several microRNAs (miRNAs) associated with PD; miR-205 suppresses LRRK2 expression and miR-205 expression levels in the brains of patients with PD decreases [16]. Furthermore, miR-34b and miR-34c are downregulated in the brains of patients with PD, which is related to the reduction in the expression of *DJ-1* and *PARKIN* [17], and miR-133 and miR-7 are also associated with PD [18–20]. Numerous reports that have described the roles of transcription factors (TFs) in PD have also been published. The TF paired-like homeodomain 3 has roles in developing and maintaining dopaminergic neurons [21, 22], and engrailed l, which is downregulated in the rat models, plays a role in the apoptosis of dopaminergic neurons and the symptoms of PD [23]. Moreover, Nrf2, nuclear factor kappa B (NF-*κ*B), GATA2, and PHD finger protein 10 are TFs involved in PD [24–27]. However, understanding the key mechanisms underlying the development of PD remains unclear.

In a previous study, the microarray dataset GSE72267 generated by Calligaris et al. [7] was used to identify key differentially expressed genes (DEGs) such as *CBX5*, *TCF3*, dedicator of cytokinesis 10, and mannosidase alpha class 1C in the blood of patients with PD compared with those of healthy controls. Moreover, crucial pathways related to chromatin remodeling and methylation were revealed. In the current study, we downloaded this microarray dataset to comprehensively analyze DEGs in patients with PD compared with those in matched controls by bioinformatics approaches and to describe their functional annotations. Compared with the previous analysis conducted by Calligaris et al. [7], we performed additional analyses, including those for the protein-protein interaction (PPI), miRNA-target regulatory, and TF-target regulatory networks, to further elucidate the key mechanisms underlying PD. Our results may provide useful data for diagnosing and treating PD.

## 2. Materials and Methods

### 2.1. Affymetrix Microarray Data

Gene expression profile data GSE72267 was extracted from the Gene Expression Omnibus database (https://www.ncbi.nlm.nih.gov/geo/) [28]. The GSE72267 dataset was deposited by Calligaris et al. [7], including blood samples from 40 PD patients and 19 healthy matched controls and was based on the platform of the GPL571 (HG-U133A-2) Affymetrix Human Genome U133A 2.0 Array (Affymetrix Inc., Santa Clara, California, USA). This dataset was downloaded and analyzed on October 2016.

### 2.2. Data Preprocessing and DEG Screening

The downloaded data in CEL files were preprocessed using the Affy package (version 1.50.0) [29] in R language, including background correction, normalization, and expression calculation. Annotations to the probes were performed, and probes that were not matched to the gene symbol were excluded. The average expression values were taken if different probes mapped to the same gene. DEGs in patients with PD compared with those in healthy matched controls were analyzed using the limma package (version 3.10.3) [30] in R language. The cutoff threshold was set to a *p* value of <0.05.

### 2.3. Pathway Enrichment Analysis

Gene ontology (GO) (http://www.geneontology.org/) analysis is commonly used for functional studies of large-scale genomic or transcriptomic data and classifies functions with respect to three aspects: molecular function (MF), cellular component (CC), and biological process (BP) [31, 32]. The Kyoto Encyclopedia of Genes and Genomes (KEGG; http://www.kegg.jp/) pathway database [33] is widely used for systematic analysis of gene functions, linking genomic data with higher order functional data. The database for annotation, visualization, and integrated discovery (DAVID) is an integrated biological knowledgebase with analytical tools used for systematic and integrative analysis of large gene lists [34]. In this study, GO terms and KEGG pathway enrichment analyses for up- and downregulated DEGs were performed using DAVID (version 6.8). The cutoff thresholds were as follows: an enrichment gene number count of ≥2 and a super geometry inspection significance threshold *p* value of <0.05.

### 2.4. PPI Network Analysis

Search Tool for the Retrieval of Interacting Genes/Proteins (STRING; http://www.string-db.org/) [35] is an online database that assesses and integrates PPIs. In this study, DEGs were mapped into the STRING database for PPI analysis, with a PPI score of 0.4 as the parameter setting. The PPI network established by DEGs was constructed using the Cytoscape software (version 3.2.0) [36], and the topology scores of the nodes, including node degree in the PPI network, were analyzed using the CytoNCA plugin (version 2.1.6; http://apps.cytoscape.org/apps/cytonca) [37] (parameter setting: without weight). Degree was used for describing importance of protein nodes in network. The higher the degree was, the more important the nodes were in network. In addition, subnetworks were identified using the MCODE plugin [38] in the Cytoscape software, and subnetworks with a score of >5 were identified as key subnetworks. Finally, KEGG pathway enrichment analyses for the genes in the key subnetworks were performed.

### 2.5. miRNA-Target Regulatory Network Analysis

The miR2disease (http://www.mir2disease.org/) database [39] is a manually curated database that provides a comprehensive resource of miRNA deregulation in various human diseases. miRWalk2.0 (http://zmf.umm.uni-heidelberg.de/apps/zmf/mirwalk2/) [40] is a comprehensive database that presents predicted and validated data, regarding miRNA targets in human, mouse, and rats. In this study, miRNAs related to PD were extracted from the miR2disease database, and experimentally verified miRNA-gene regulatory pairs were obtained by searching miRWalk2.0. Finally, a miRNA-target regulatory network was constructed by comparing DEGs with obtained miRNA-gene regulatory pairs using the Cytoscape software.

### 2.6. TF-Target Regulatory Network Analysis

The genes in the PPI network described above were further analyzed to identify TF-target interaction pairs that were then used to construct a TF-target regulatory network. The iRegulon plugin (version 1.3; http://apps.cytoscape.org/apps/iRegulon) [41] in the Cytoscape software collects multiple human TF-target interaction databases such as Transfac, Jaspar, and Encode using two computational methods: Motif and Track. In this study, we analyzed the TF-target pairs using the iRegulon plugin and compared them with TFs with DEGs in the PPI network, followed by a TF-target regulatory network construction. The parameter settings were as follows: minimum identity between orthologous genes, 0.05 and maximum false discovery rate on motif similarity, 0.001. The normalized enrichment score (NES) indicates the reliability of the results, and the cutoff threshold was NES of >3.

## 3. Results

### 3.1. Analysis of DEGs

**Figure 1:**
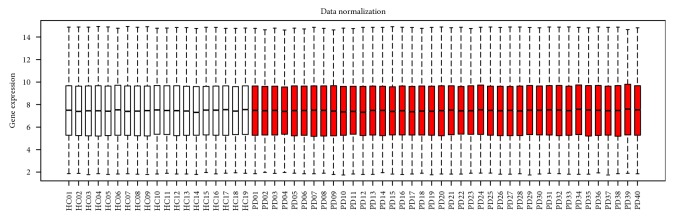
Boxplots for normalized gene expression data. Red represents the blood samples of patients with Parkinson's disease, and white represents the healthy matched control samples.

The boxplot of the preprocessed data indicated good normalization (Figure 1). In total, 22,277 probes were obtained, among which 971 probes were differentially expressed. After annotation, 819 DEGs in patients with PD compared with those in healthy matched controls were identified (Supplementary Table 1), including 359 upregulated DEGs and 460 downregulated DEGs.

### 3.2. Pathway Enrichment Analysis

**Figure 2:**
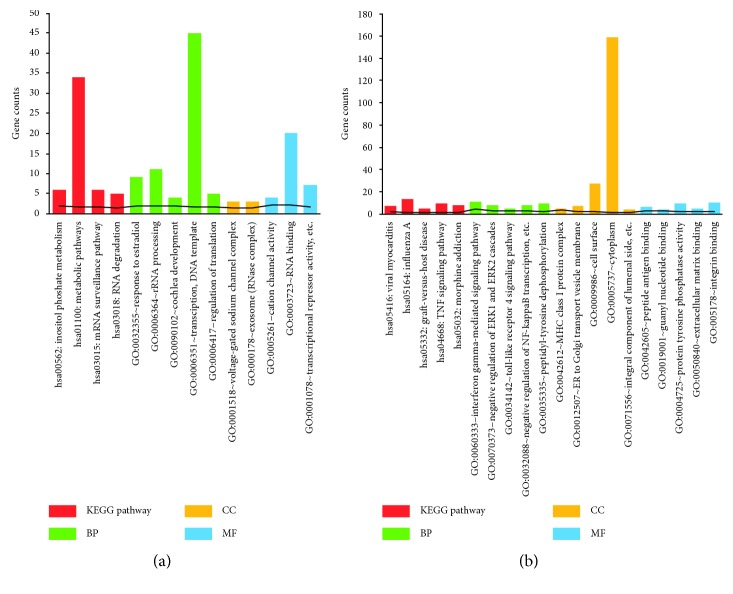
Functional enrichment analyses of differentially expressed genes (DEGs). (a) Gene Ontology (GO) terms and the Kyoto Encyclopedia of Genes and Genomes (KEGG) pathways of upregulated DEGs and (b) GO terms and KEGG pathways of downregulated DEGs. The numbers on the *x*-axis were the ID of pathways or GO terms. The numbers on the *y*-axis were gene counts.

GO and KEGG pathway enrichment analyses for the up- and downregulated DEGs were performed (Supplementary Table 2). The significant GO terms and KEGG pathways are shown in Figure 2. The upregulated DEGs were significantly enriched in four KEGG pathways, namely, metabolic pathways, inositol phosphate metabolism, mRNA surveillance pathway, and RNA degradation, and GO terms such as transcription, DNA-template processing, and rRNA processing (Figure 2). The downregulated DEGs were enriched in pathways such as those of influenza A, viral myocarditis, and TNF signaling and GO terms such as cytoplasm, cell surface, and interferon gamma-mediated signaling pathway (Figure 2).

### 3.3. PPI Network Analysis

**Figure 3:**
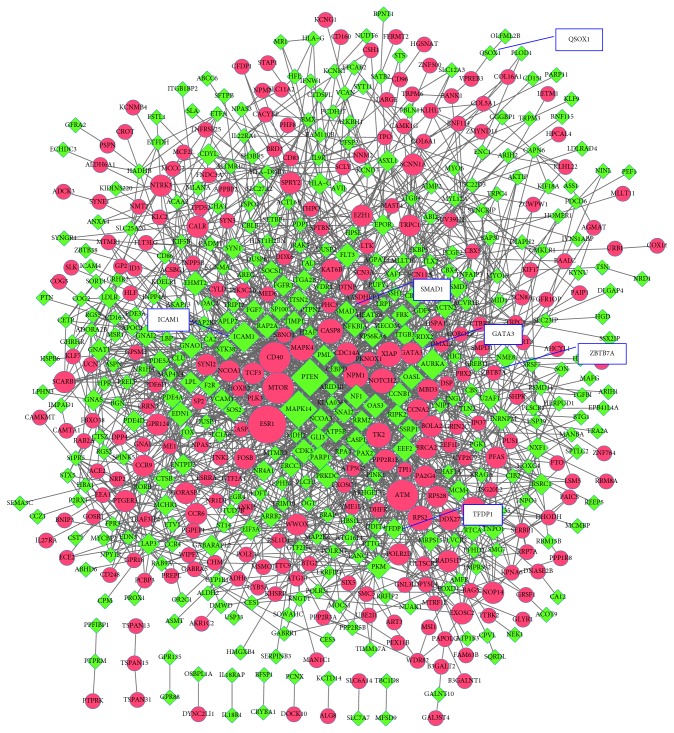
The protein-protein interaction (PPI) network of differentially expressed genes (DEGs). Red circles represent upregulated DEGs, and green diamonds represent downregulated DEGs.

**Figure 4:**
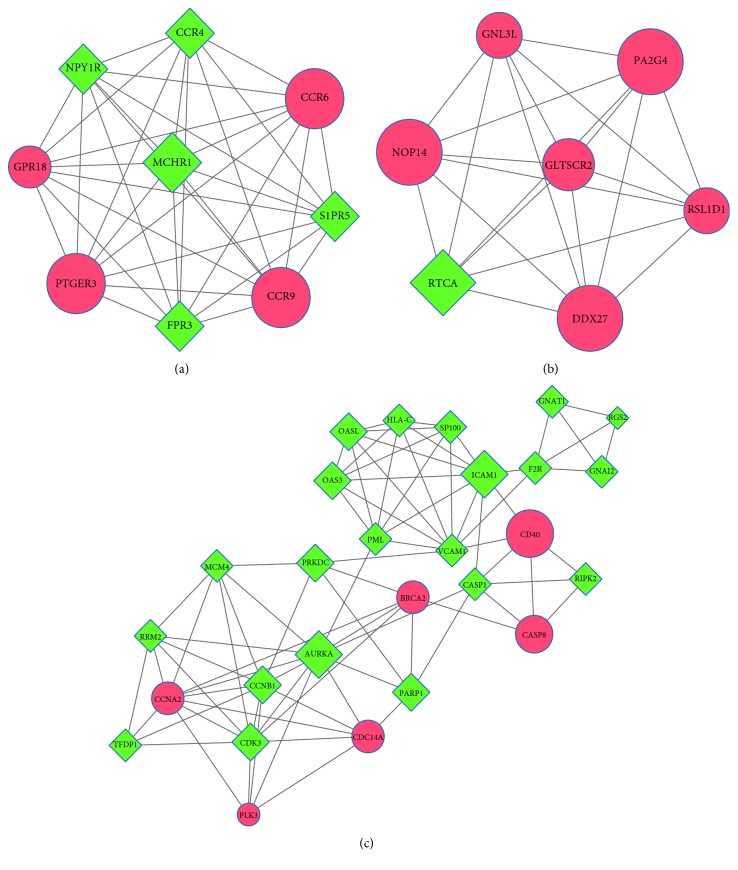
Subnetworks of differentially expressed genes (DEGs). (a) Subnetwork a; (b) subnetwork b; (c) subnetwork c. Red circles represent upregulated DEGs, and green diamonds represent downregulated DEGs.

The PPI network, including 605 nodes and 1937 PPI pairs, is shown in Figure 3. The top 10 DEGs with the highest degree included five upregulated DEGs such as estrogen receptor 1 (*ESR1*), mechanistic target of rapamycin (*MTOR*), ATM serine/threonine kinase (*ATM*), CD40 molecule (*CD40*) and thymidine kinase 2, mitochondrial (*TK2*), and five downregulated DEGs such as mitogen-activated protein kinase 14 (*MAPK14*), phosphatase and tensin homolog (*PTEN*), intercellular adhesion molecule 1 (*ICAM1*), aurora kinase A (*AURKA*), and protein kinase, DNA-activated, catalytic polypeptide (*PRKDC*) (Table 1). Three subnetworks were identified (subnetworks a–c). Subnetwork a (Figure 4) included nine nodes and 36 PPI pairs, and these genes were significantly enriched in three KEGG pathways (Table 2), including neuroactive ligand-receptor interaction, chemokine signaling pathway, and cytokine-cytokine receptor interaction. Subnetwork b (Figure 4) included seven nodes and 21 PPI pairs, and these genes were not enriched in any KEGG pathway. Subnetwork c (Figure 4) included 27 nodes and 81 PPI pairs, and these genes were enriched in 12 KEGG pathways (Table 2), such as cell cycle, herpes simplex infection, and NF-*κ*B signaling pathways. In addition, *ICAM1* was involved in six KEGG pathways of subnetwork c, such as viral myocarditis, cell adhesion molecules (CAMs), and NF-*κ*B signaling pathways (Table 2). The detailed information existed in PPI network, and three subnetworks are shown in Supplementary Table 3.

### 3.4. miRNA-Target Regulatory Network Analysis

**Figure 5:**
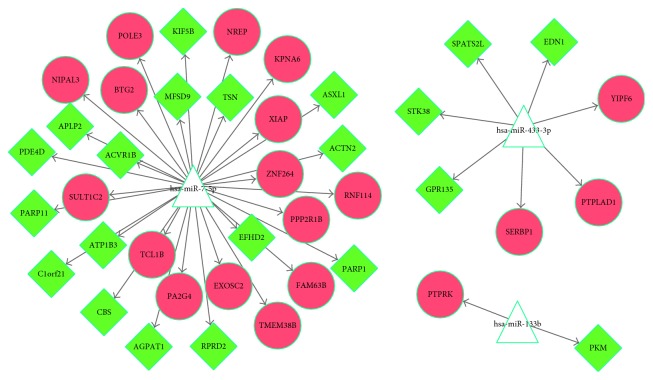
MicroRNA- (miRNA-) target regulatory networks of differentially expressed genes (DEGs). Triangles represent miRNAs, red circles represent upregulated DEGs, and green diamonds represent downregulated DEGs.

According to the data from the miR2disease database, six miRNAs were identified to be associated with PD and 698 miRNA-gene pairs were obtained by searching miRWalk2.0. A total of 40 miRNA-target interaction pairs were obtained by comparing miRNA-gene pairs with DEGs, and subsequently, the miRNA-target regulatory network was constructed. The network (Figure 5) contained 40 miRNA-target interaction pairs and 43 nodes (Supplementary Table 4), among which three miRNAs (hsa-miR-7-5p, hsa-miR-433-3p, and hsa-miR-133b) were included.

### 3.5. TF-Target Regulatory Network Analysis

**Figure 6:**
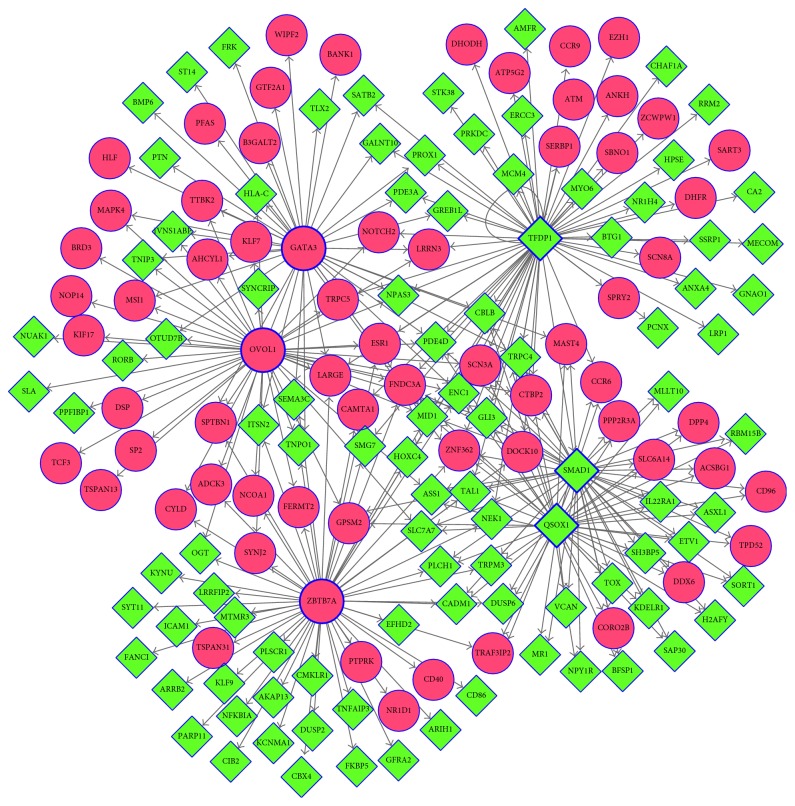
The TF-target regulatory network of differentially expressed genes (DEGs). Blue boxed figures represent TFs, red circles represent upregulated genes, and green diamonds represent downregulated genes. TF, transcription factor.

According the information of TF-target interaction databases such as Transfac, Jaspar, and Encode in the Cytoscape software, a total of 83 TFs were identified from the PPI network, forming 5371 TF-gene pairs. Among the 83 TFs, six were differentially expressed: three upregulated ones, that is, zinc finger and BTB domain-containing 7A (*ZBTB7A*), ovo-like transcriptional repressor 1 (*OVOL1*), and GATA-binding protein 3, and three downregulated ones, that is, transcription factor dp-1 (*TFDP1*), SMAD family member 1 (*SMAD1*), and quiescin sulfhydryl oxidase 1 (*QSOX1*). The TF-target regulatory network (Figure 6) was constructed and included 166 nodes and 288 interaction pairs (Supplementary Table 5). The top 20 nodes with the highest degree are listed in Table 3, including the six TFs described above and 14 other DEGs, such as ectodermal-neural cortex 1, fibronectin type III domain-containing 3A, and midline 1, which were coregulated by the six TFs.

## 4. Discussion

PD is the second most common age-related neurodegenerative disease. However, the pathogenesis and genes involved in PD are not well known [42]. In this study, we performed a comprehensive bioinformatics analysis of the blood gene expression profile using the GSE72267 dataset. The results suggested that four key pathways (metabolic pathways, TNF signaling pathway, rRNA processing, and cytoplasm), the key gene *ICAM1*, three miRNAs (hsa-miR-7-5p, hsa-miR-433-3p, and hsa-miR-133b), and six TFs (*ZBTB7A*, *OVOL1*, *GATA3*, *TFDP1*, *SMAD1*, and *QSOX*) might play important roles in PD development.

Our results revealed that the upregulated DEGs were enriched in the KEGG pathway “metabolic pathways” and the GO term “rRNA processing,” and the downregulated DEGs were enriched in the KEGG pathway “TNF signaling pathway” and the GO term “cytoplasm.” A previous study [43] demonstrated that some metabolic patterns were altered in patients with advanced PD. Multiple metabolic pathways are also involved in PD [44], which supports our study results. Cytoplasmic inclusions are a pathological hallmark of PD [45]. Lewy body pathology is involved [46, 47], and glial cytoplasmic inclusions are associated with Lewy bodies [48]. Thus, the GO term “cytoplasm” may play a role in PD. Furthermore, TNF receptor-associated protein is excluded from the nucleolus and is sequestered to the cytoplasm by TNF receptor-associated factor 6, thereby altering ribosomal RNA (rRNA) biogenesis [49]. The TNF signaling pathway is also involved in PD [50], and rRNA transcription is repressed in patients with PD [51]. Therefore, the GO term “rRNA processing” and the KEGG pathway “TNF signaling pathway” may play important roles in PD. Altogether, the metabolic pathways, TNF signaling pathway, rRNA processing, and cytoplasm are essentially involved in PD pathogenesis.

*ICAM1* was among the top 10 DEGs in the PPI network. Moreover, *ICAM1* gene was involved in six KEGG pathways for subnetwork c. *ICAM1* is involved in the adhesion and transmigration of leukocytes across the endothelium, promoting brain inflammation and resulting in brain diseases [52]. T helper 17 cells can exert a neurotoxic effect in the brain parenchyma of patients with PD by interacting with *ICAM1* and leukocyte function-associated antigen 1 [53]. In addition, *ICAM1* is involved in persistent inflammation in PD [54]. Our results from the KEGG pathway analysis for genes in subnetworks revealed that *ICAM1* might play roles in viral myocarditis and CAMs and thus contributed to PD.

The miRNA-target regulatory network analysis identified three miRNAs involved in PD, namely, hsa-miR-7-5p, hsa-miR-433-3p, and hsa-miR-133b. A study described miR-7-2 dysregulation (the stem loop of hsa-miR-7-5p) in Parkinson's patient's leukocytes [55] and revealed that hsa-miR-7-5p expression decreased in PD, possibly upregulating *α-SYN*, a PD-related gene [56]. The variation of the hsa-miR-433- (the stem loop of hsa-miR-433-3p-) binding site of fibroblast growth factor 20 can lead to *α-SYN* overexpression, increasing the risk for PD [57]. hsa-miR-133b expression is increased in the cerebrospinal fluid of patients with PD [58]; however, its expression levels in serum is decreased, which is related to low serum ceruloplasmin levels [59]. hsa-miR-133b is also deficient in the midbrain tissue of patients with PD and is associated with the maturation and function of midbrain dopaminergic neurons [60]. Notably, reduced circulating levels of miR-433 and miR-133b are considered as promising biomarkers for PD [61]. Therefore, we speculate that the three miRNAs, including hsa-miR-7-5p, hsa-miR-433-3p, and hsa-miR-133b may play important roles in PD.

TFs are important regulators of target gene expressions [53, 62]. In this study, we analyzed DEGs in the PPI network to screen TFs involved in PD. Among the 83 TFs identified in the PPI network, six were found to be differentially expressed. *ZBTB7A*, *OVOL1*, and *GATA3* were upregulated in patients with PD compared with those in healthy matched controls, whereas *TFDP1*, *SMAD1*, and *QSOX1* were downregulated. *ZBTB7A* is a tumor suppressor, which is involved in several cancers such as prostate and nonsmall cell lung cancers [63–65]. *OVOL1*, encoding a zinc finger protein, is expressed in embryonic epidermal progenitor cells and is an inducer of mesenchymal-to-epithelial transition in human cancers [66, 67]. *GATA3*, a member of the GATA family, is a regulator of T-cell development and plays roles in endothelial cells [68, 69]. *TFDP1* is involved in the cell cycle and contributes to hepatocellular carcinomas [70, 71], *SMAD1* is involved in multiple pathways [72, 73], and *QSOX1* plays roles in some cancers such as breast cancer and neuroblastoma [74–76]. However, there are few reports regarding the involvement of these TFs in PD. Hence, further studies regarding the associations between the TFs identified in this study and PD are warranted.

In conclusion, our data demonstrated that the metabolic pathways, TNF signaling pathway, rRNA processing, and cytoplasm play important roles in PD pathogenesis; *ICAM1* might also play a vital role. Besides six TFs, three miRNAs, including hsa-miR-7-5p, hsa-miR-433-3p, and hsa-miR-133b, may be involved in PD. However, because of the study limitations, further investigation remains to be performed in the future.

## Figures and Tables

**Table 1:** List of top 10 differentially expressed genes with higher degrees in protein-protein interaction network.

Gene	Full name	Description	Degree
MAPK14	Mitogen-activated protein kinase 14	Down	68
ESR1	Estrogen receptor 1	Up	54
PTEN	Phosphatase and tensin homolog	Down	52
MTOR	Mechanistic target of rapamycin	Up	40
ATM	ATM serine/threonine kinase	Up	35
ICAM1	Intercellular adhesion molecule 1	Down	33
CD40	CD40 molecule	Up	32
AURKA	Aurora kinase A	Down	31
PRKDC	Protein kinase, DNA-activated, catalytic polypeptide	Down	29
TK2	Thymidine kinase 2, mitochondrial	Up	29

Degree was used for describing the importance of protein nodes in network. The higher the degree was, the more important the nodes were in network.

**Table 2:** List of KEGG pathways of subnetworks.

Subnetwork	Pathway ID	Pathway name	Count	*p* value	Genes
Subnetwork a	hsa04080	Neuroactive ligand-receptor interaction	5	1.40*E*−04	MCHR1, PTGER3, S1PR5, FPR3, NPY1R
	hsa04062	Chemokine signaling pathway	3	1.80*E*−02	CCR9, CCR6, CCR4
	hsa04060	Cytokine-cytokine receptor interaction	3	2.74*E*−02	CCR9, CCR6, CCR4
Subnetwork c	hsa04110	Cell cycle	6	1.31*E*−04	CCNB1, CDC14A, PRKDC, CCNA2, MCM4, TFDP1
	hsa05416	Viral myocarditis	4	1.62*E*−03	ICAM1, CASP8, HLA-C, CD40
	hsa05168	Herpes simplex infection	5	6.03*E*−03	SP100, CASP8, OAS3, PML, HLA-C
	hsa04514	Cell adhesion molecules	4	1.70*E*−02	VCAM1, ICAM1, HLA-C, CD40
	hsa05144	Malaria	3	1.78*E*−02	VCAM1, ICAM1, CD40
	hsa04621	NOD-like receptor signaling pathway	3	2.00*E*−02	CASP8, RIPK2, CASP1
	hsa04115	p53 signaling pathway	3	2.93*E*−02	CCNB1, RRM2, CASP8
	hsa05164	Influenza A	4	3.32*E*−02	ICAM1, OAS3, PML, CASP1
	hsa04914	Progesterone-mediated oocyte maturation	3	4.30*E*−02	CCNB1, GNAI2, CCNA2
	hsa05169	Epstein–Barr virus infection	4	4.42*E*−02	ICAM1, HLA-C, CD40, CCNA2
	hsa05203	Viral carcinogenesis	4	4.60*E*−02	SP100, CASP8, HLA-C, CCNA2
	hsa04064	NF-kappa B signaling pathway	3	4.84*E*−02	VCAM1, ICAM1, CD40

KEGG, Kyoto Encyclopedia of Genes and Genomes.

**Table 3:** List of top 20 nodes with higher degree in transcription factor-target regulatory network.

Gene	Full name	Description	Degree
TFDP1^∗^	Transcription factor Dp-1	Down	62
ZBTB7A^∗^	Zinc finger and BTB domain-containing 7A	Up	55
OVOL1^∗^	Ovo-like transcriptional repressor 1	Up	46
SMAD1^∗^	SMAD family member 1	Down	45
QSOX1^∗^	Quiescin sulfhydryl oxidase 1	Down	44
GATA3^∗^	GATA-binding protein 3	Up	38
ENC1	Ectodermal-neural cortex 1	Down	6
FNDC3A	Fibronectin type III domain-containing 3A	Up	6
MID1	Midline 1	Down	6
PDE4D	Phosphodiesterase 4D	Down	5
ZNF362	Zinc finger protein 362	Up	5
CBLB	Cbl proto-oncogene B	Down	4
LARGE	LARGE xylosyl- and glucuronyltransferase	Up	4
TRPC4	Transient receptor potential cation channel subfamily C member 4	Down	4
CTBP2	C-terminal binding protein 2	Up	4
GLI3	GLI family zinc finger 3	Down	4
SCN3A	Sodium voltage-gated channel alpha subunit 3	Up	4
TAL1	TAL BHLH transcription factor 1, erythroid differentiation factor	Down	4
LRRN3	Leucine rich repeat neuronal 3	Up	3
MAST4	Microtubule-associated serine/threonine kinase family member 4	Up	3

^∗^Transcription factor.

## Supplementary Materials

Supplementary 1 Table 1: all up- and downregulated differentially expressed genes.

Supplementary 2 Table 2: GO and KEGG pathway enrichment analyses for the up- and downregulated differentially expressed genes. GO, Gene Ontology; KEGG, Kyoto Encyclopedia of Genes and Genomes.

Supplementary 3 Table 3: the detailed information existing in protein-protein interaction (PPI) network and three subnetworks.

Supplementary 4 Table 4: the detailed information about the miRNA-target regulatory network.

Supplementary 5 Table 5: the detailed information about transcription factor- (TF-) target regulatory network.
